# Optimizing the Use of a Precious Resource: The Role of Emergency Physicians in a Humanitarian Crisis

**DOI:** 10.5811/westjem.2017.3.32718

**Published:** 2017-04-19

**Authors:** Parveen K. Parmar, P. Gregg Greenough

**Affiliations:** * University of Southern California, Department of Emergency Medicine, Division of International Emergency Medicine, Los Angeles, California; † Harvard Medical School, Department of Emergency Medicine, Division of International Emergency Medicine and Humanitarian Programs, Cambridge, Massachusetts; ‡ Brigham and Women’s Hospital, Department of Emergency Medicine, Boston, Massachusetts

## Abstract

Emergency physicians (EP) are uniquely suited to provide care in crises as a result of their broad training, ability to work quickly and effectively in high-pressure, austere settings, and their inherent flexibility. While emergency medicine training is helpful to support the needs of crisis-affected and displaced populations, it is not in itself sufficient. In this article we review what an EP should carefully consider prior to deployment.

## INTRODUCTION

You wake up at dawn to get ready for a day shift. As you’re making coffee and preparing a quick breakfast, you turn on the news. An earthquake measuring 7.0 on the Richter scale has hit Haiti, resulting in chaos, tens of thousands of deaths and injuries. The local healthcare system is overwhelmed, and the pictures are devastating.

While heading to work, you feel deeply moved as you contemplate the suffering in Haiti. As an emergency physician, you are compelled to help. You reason that with only a few shifts to work in the coming two weeks, you could easily move these to deploy and provide assistance. But you have never helped in a crisis response before. You know you want to help in the most impactful way possible but aren’t sure what to do.

Emergency physicians (EP) are uniquely suited to provide care in crises as a result of their broad training, ability to work quickly and effectively in high-pressure, austere settings, and their inherent flexibility. A board-certified EP is comfortable treating a trauma victim in one room, complications of pregnancy in another, and geriatric or pediatric emergencies in another. Additionally, EPs are well versed in managing acute complications of non-communicable diseases such as diabetes and hypertension, an increasingly common problem in recent crises as a result of the global burden of disease shifts with improving development.

While emergency medicine (EM) training is helpful to support the needs of crisis-affected and displaced populations, it is not in itself sufficient. In this article we review what an EP should carefully consider prior to deployment. This article will not consider the increasing role EPs play in the development of EM abroad as educators and clinicians. However, many of the principles discussed below apply in that context as well.

## ALL AID IS NOT HELPFUL

EPs often wish to assist in the days and weeks following an emergency, but they may have less than 2–3 weeks time available. Often, with the best of intentions, they deploy with whatever group will take them for the short term, or they simply take a flight and arrive, hoping to find a way to help upon arrival. This is a dangerous practice for many reasons.

The influx of a large number of relatively inexperienced individuals and organizations into a crisis area—academic medical centers with limited global health capacity, faith-based groups, or private organizations have all been culprits—can ultimately cause more harm than good. The 2010 Haiti earthquake was a clear recent example of this. Given physical proximity to the United States, a large number of medical groups and organizations that had neither an understanding of a low-income health system nor the knowledge of working within the international humanitarian community’s framework in complex emergencies intervened in Haiti with poor results.^1^ The Inter-Agency Standing Committee, a forum for coordination, policy, and development comprised of United Nations operational groups, the International Committee and Federation members of the Red Cross and Red Crescent Movement and non-governmental organizations (NGOs), published a review of the humanitarian response that highlighted the negative impact of this impulsive intervention. Their findings suggest that less-experienced groups do not understand the existing U.N.-based coordination mechanisms in humanitarian response, namely the cluster approach for humanitarian coordination.^2^ Less-experienced organizations often did not participate in this cluster system resulting in poor coordination and overlapping or competing efforts that led to waste of limited resources and general chaos.^3^

Public Radio International highlighted the problems specifically created by physicians who flocked to Haiti without adequate training or supplies, minimal previous humanitarian experience, and either poor or no backing by established humanitarian organizations.^4^,^5^ Surgeons arrived without either bringing anesthesia or ensuring that it was available in country. As one seasoned, humanitarian EP described during the interview: “Surgeries were either delayed because surgeons didn’t want to operate without the anesthesia, or people had to undergo amputations and other surgeries without anesthesia, which was horrifying.” Other surgeons found anesthesia and other medications had expired or no longer worked as a result of improper storage. Smaller, inexperienced groups of providers had done minimal-to-no needs assessments (perhaps because they did not know how), did not coordinate with any formal U.N. mechanisms, had poor supply chains and storage, and ultimately were unable to provide a net positive impact in the region. Even worse, when these groups left, thousands of surgical patients remained without adequate post-operative follow-up, overwhelming local health systems.

As a result, some national and international humanitarian experts have suggested that future responses implement an airport-based screening process for NGOs, only allowing those NGOs with adequate experience, training, and credentials permission to pass through customs during times of crisis.^3^ Similarly, EPs and others wishing to assist in a crisis should ensure that they have adequate organizational backing *and* humanitarian training to help, rather than hinder, the response.

## HOW TO ENGAGE IN HUMANITARIAN AID RESPONSIBLY

An EP’s broad medical knowledge base, coupled with the ability to co-manage different types of patients and priorities, work in chaotic and uncertain environments and generally having flexible schedules, make EM one of the specialties most sought after by humanitarian organizations.^6^ However, several principles must be adhered to in order to ensure that EPs engage in humanitarian aid in a positive manner and minimize harm. Prior to deploying, ask yourself the following questions.

### Are you appropriately trained to provide care in a humanitarian crisis?

Despite a broad medical training, EPs may not be well prepared to care for the types of illnesses seen in a crisis context or to manage common illnesses in a severely resource-limited context. Clinicians should be familiar with health delivery standards in emergency settings, the disease processes that are commonly seen in the affected country, and the critical public health interventions that are foundational to all humanitarian populations. The average U.S.-based EP has likely not seen malaria, acute malnutrition or cholera, or appreciated the devastating consequences of missing key vector, water and air-borne diseases in temporary settlements.

Measles, cholera, bacterial and parasitic diarrhea, acute respiratory tract illness, malaria, and other infectious diseases are often seen during the acute phase of a crisis. As a result, immediate priorities in an emergency include measles vaccination and adequate provision of water and sanitation, food and nutrition.^7^ Post emergency-phase priorities later broaden to include reproductive health, HIV and tuberculosis programs, and psychosocial and mental health needs. Reproductive health needs may be complicated by high rates of sexual violence in certain settings.

A globally aging population and increased rates of obesity, smoking, and poor dietary habits have also led to a dramatic increase in the burden of non-communicable disease in low- and middle-income countries.^8^ As such, new conflict-affected populations reflect this: Refugees from Syria, Iraq and Afghanistan suffer from high rates of diabetes, hypertension, obesity and smoking and often present to NGO health facilities with complications of untreated non-communicable diseases, presenting clinicians with the unique challenge of managing resource-intensive chronic illnesses in resource-limited settings.^9^–^13^ These priorities are reinforced in the internationally recognized “Sphere standards,” which provide expert, consensus-based best practices for delivery of services in humanitarian crises, including both natural disasters and conflict.^14^

Thus, EPs deploying to crisis settings need to be well versed not only in traditional infectious disease needs of displaced populations, trauma management, and reproductive health, sexual violence and mental health needs, but in the management of non-communicable diseases as well – and understand how these health needs can be most appropriately and sustainably treated in a crisis setting.

Typically, the scope of medical practice in humanitarian populations requires dedicated training, which most well-established humanitarian organizations offer after one has been accepted by the organization and prior to field deployment.^15^,^16^ Humanitarian studies courses, ranging from a few weeks to year or two-year masters’ degrees with humanitarian tracks, are available at many institutions.^17^ The table provides useful field texts on health issues in crises, several of which are online or field portable.

### Do you understand how humanitarian aid works?

**Figure 1:**
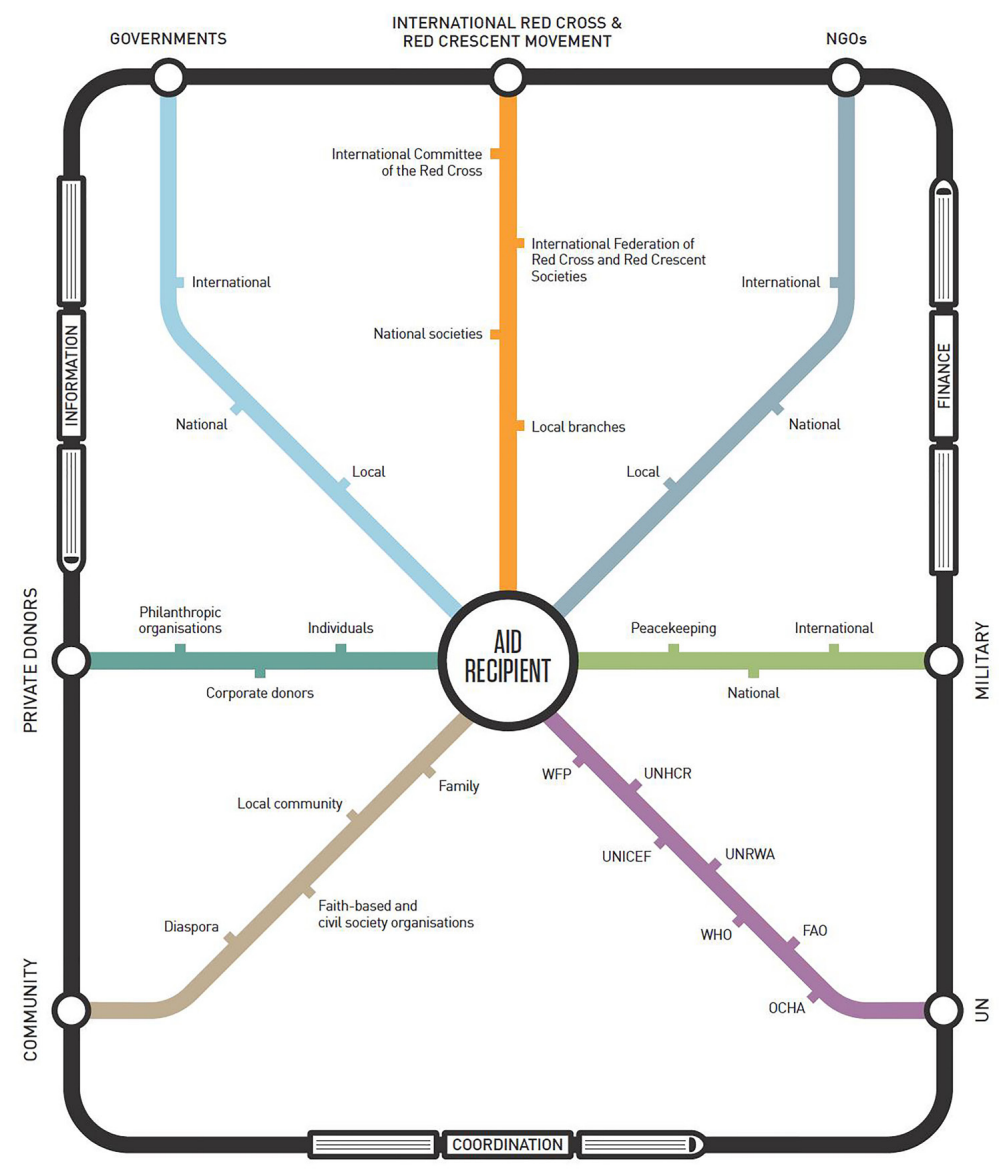
Components of the humanitarian aid network, including international organizations, non-governmental organizations (NGOs), private donors, military actors, and community members: An overview of the ways in which these components interact.^34^ *WFP,* World Food Programme; *UNHCR,* United Nations High Commissioner for Refugees; *UNICEF,* United Nations Children’s Fund; *UNRWA,* United Nations Relief and Works Agency; *WHO,* World Health Organization; *FAO,* Food and Agriculture Organization; *OCHA,* Office for the Coordination of Humanitarian Affairs.

Many stakeholders participate in humanitarian response, each with defined perspectives, roles, missions and mandates (Figure 1). Over the past several decades, humanitarian practitioners have made extensive strides towards professionalization of the field. Practitioners should understand the well-developed systems that exist for the delivery of care in crisis settings prior to deployment. At the outset of a crisis, the U.N. Office for the Coordination of Humanitarian Affairs (UN-OCHA) determines the type of response needed and may decide to declare the highest level (Level 3) emergency, immediately mobilizing funds and requesting the assistance of international health NGOs (such as Médicins Sans Frontieres (MSF), International Rescue Committee, International Medical Corps, Médicins du Monde, Mercy Corps, Save the Children, CARE, and the International Committee of the Red Cross/Red Crescent, among others). The coordinating mechanism has established protocols to conduct rapid needs assessments in the immediate aftermath of a crisis to ensure aid is efficient and targets those with the greatest needs. All international and local health organizations are expected to coordinate with UN-OCHA and its modular “cluster system”; 18 the health cluster, coordinated by the World Health Organization (WHO), meets regularly throughout a crisis to ensure all health agencies are coordinating, sharing assessment data and program information about areas of greatest need, and distributing aid in a way that avoids duplication or misses populations in need. Additional “clusters” coordinate agencies concerned with water and sanitation, shelter, nutrition, and protection, among others; many of these directly impact population health outcomes.

Several resources that discuss humanitarian coordination mechanisms and standards for delivery of care are available online and free of charge. These include the “Building a Better Response” course (http://www.buildingabetterresponse.org/), which outlines the U.N. cluster system and the importance of coordination, and disasterready.org, a free online resource developed by a coalition of prominent humanitarian organizations that offers a range of courses with topics entitled Humanitarianism, Program/Operations, Protection, Staff Welfare, Management and Leadership, and Staff Safety & Security. Finally, the Sphere standards referenced above^14^ should be familiar to all clinicians deploying to a crisis setting.

The best way to ensure adequate preparation is to deploy with a well-established, reputable humanitarian organization with experience in preparing volunteers and employees prior to deployment. Below, we outline some key issues to consider in evaluating an organization.

### Should I deploy with this organization?

Well-established humanitarian NGOs have a long history of working in crisis settings, require training of all deployed clinicians and field workers, and generally require a minimum deployment of a month or more. 19 These organizations will have well-developed security and evacuation protocols, medical insurance for field workers, human resources departments, logistics expertise, and a history of working effectively with U.N. coordination mechanisms, national ministries of health, as well as other local and international NGOs.

Staff with these organizations may have professional degrees in humanitarian aid, and are familiar with international resources and standards for the provision of aid such as the aforementioned Sphere standards and WHO emergency health kits.^20^ They are intimately familiar with the ways in which health needs interact with food and nutrition, water and sanitation, and other sector systems that are disrupted after a disaster or conflict.

**Figure 2:**
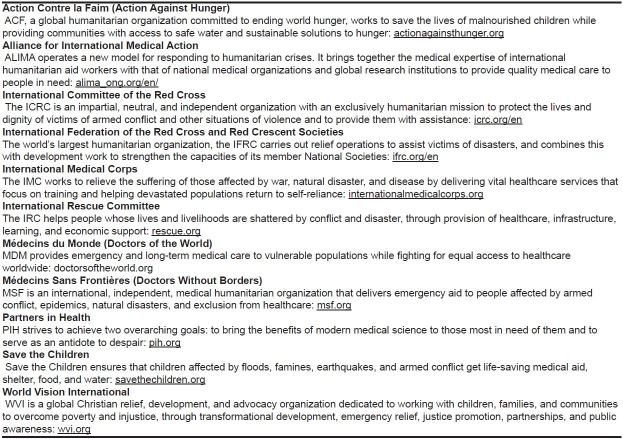
Select list of reputable organizations involved in healthcare delivery. *From Lampard B, et al. Global Humanitarian Medicine and Disaster Relief. In: *Auerbach’s Wilderness Medicine*.7th ed. Philadelphia, PA. Elsevier; 2012:1893–1928.

If you decide that deploying is the right decision for you, it is critical to obtain relevant humanitarian training and deploy with a reputable organization that understands the principles involved in working in a complex emergency and that engages in local coordination mechanisms. A partial list of well-established humanitarian organizations is in Figure 2. These organizations will typically have rosters for clinicians to join and often require pre-deployment training. If you are interested in deploying, take the opportunity to speak with these organizations, get on their rosters, get trained, and deploy during a future crisis.

**Figure 3:**
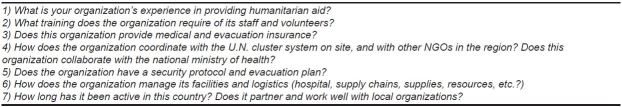
Questions to ask to determine whether or not an organization is well-prepared for a humanitarian response.

Ask questions of the organization prior to signing up; a list is provided below (Figure 3). Often the best way to determine the reputation of an organization is to ask experienced colleagues, in particular those who have worked with the organization with whom you wish to deploy. Many major academic centers have international EM faculty who have worked closely with U.N. operational agencies and humanitarian NGOs and can provide advice.

### Do you speak the language?

These health needs and the settings for healthcare provision often occur in highly complex political and social settings where English is not the primary language spoken. Clinicians routinely overestimate their fluency.^21^ Even with non-English language fluency, practitioners may struggle with dialects that vary significantly from country to country or may not be familiar with medical terminology. During crises, already overburdened health staff are typically overwhelmed; thus, you cannot assume local support staff will have the time (or skills) to provide adequate interpretation. Ensure either that you speak the language prior to deployment or that adequate, dedicated interpreter services exist for you when you arrive—and ensure interpreter services are skilled at providing medical interpretation. Failing to do so can create additional burdens on an already under-resourced system.

### Do you have enough time?

Physicians should ensure they have enough time to familiarize themselves with local medications, health systems, local colleagues, and the medical context in order to be useful. Specifically, this requires knowledge of the population demographic; the state of the health system at the national, district and local levels and how it functions at each; endemic diseases with knowledge of local vectors; and the country’s baseline burden of communicable and non-communicable diseases. Experienced clinicians with pre-existing humanitarian and local expertise can be helpful in the short term, but this is generally the exception and not the rule.

As noted above, a two-week deployment is typically insufficient even for experienced clinicians. Consider that resources spent on short-term responses of questionable value might have paid for several full-time clinicians to work on site for several months, or for appropriate medical supplies, food aid, etc. Money spent on travel may be of much more use as cash aid to reputable organizations providing aid.^22^ For example, according to the MSF website, a donation of U.S. $35 provides two high-energy meals for 200 malnourished children for a day; U.S. $50 can provide vaccinations for meningitis, measles, and polio for 50 people; U.S. $100 can provide antibiotics for 40 children; and a donation of U.S. $1000 can provide emergency medical supplies to 5,000 disaster victims for a month.^23^ Considering that the cost of a plane ticket, room and board is roughly $2,000–$5,000 depending on the location of the crisis, direct financial support to professional organizations in disaster response almost always has a higher impact than deploying for a few weeks.

That being said, several models for long-term, sustainable engagement in global health are available to EPs.

## MODELS FOR SUSTAINABLE ENGAGEMENT IN HUMANITARIAN AID

Below are a few models the authors have encountered that allow U.S.-based EPs to engage sustainably in humanitarian aid.

### A note about trainees

Deployment of trainees (medical students, residents) to a humanitarian crisis zone is rarely appropriate, as there are not enough resources or time to allow for adequate supervision of residents or medical students in a crisis. Trainees engaging in unsupervised clinical practice abroad is largely viewed as unethical,^24^–^26^ and can put academic institutions at risk. International EM fellows are often deployed to crisis zones, but generally have had significant training in standards and best practices in humanitarian aid prior to travel. Residents and students with an interest in humanitarian aid are advised to seek mentorship from experienced humanitarians and members of academic international EM departments on how they might best prepare themselves for work in humanitarian crises once their training is complete. Several opportunities exist to support domestic refugees and asylum seekers,^27^ and trainees can lead very successful fundraising and awareness campaigns in their local communities.

### A full time humanitarian career

Some EPs choose to enter a career as a full-time humanitarian aid worker. While this is often done immediately after residency, it is an option for the mid- or late-career EPs as well. This will typically involve a 6–9 month initial deployment, with the option to stay on at that site or deploy to another crisis. One can apply online or speak to a recruiter for the organizations listed in Figure 2 to explore available options.

The process generally involves a written application and interview, and if selected, requires training and deployment shortly afterwards. Depending on the organization, one may or may not have the option of choosing one’s initial field site. Deployments are generally to austere settings with varying access to phone, internet, and other Western comforts. Some sites may be in or near active conflict zones.

Exposure to trauma, caring for survivors of war, witnessing human rights violations and living in insecure, poor settings can be difficult. Aid workers themselves can be the target of violence and locally transmitted infectious diseases, and their mental health can suffer.^28^ Not every clinician is suited to this work. However, full-time humanitarians have the greatest impact in the field, amassing a wealth of knowledge and experience and developing close relationships with those residing in country as well as their fellow humanitarians. Most would describe their work as deeply rewarding despite the challenges.

Financial considerations often keep EPs from deploying full time. According to a recent study, the average EM resident has $212,000 of educational debt, roughly 25% more than the average mortgage in the U.S.^29^ Humanitarians are not generally paid enough to make payments on these loans; however, loan deferment can be sought while deployed.

### Regular deployments and community EM practice

Given the above-mentioned financial constraints, some EPs divide their time between working in humanitarian crises and working in community emergency departments. While financially more sustainable, this can be challenging. First, it may be difficult to find a clinical practice that allows for the many months required in the field. EPs sometimes opt for locum tenens positions during periods of non-deployment, which allows for flexibility and short-term clinical practice while in the U.S. Others find departments with low overall shift requirements and flexible scheduling policies that allow them to batch shifts while in country and travel for 1–2 months at a time—though this arrangement does not allow for 3–9 month field requirements. Keep in mind, however, that many organizations will agree to shorter deployments once you have worked with them for several longer deployments—allowing you to spend more time in your home country as you become more experienced.

Some departments might be willing to consider splitting a full-time position in half, allowing you and a colleague to work six months a year, and deploy to the field for six months. It is critical to engage the support of department leadership to ensure that their needs are met when seeking these alternative arrangements, and to be a good citizen of the department once the arrangement has been made (ensure charts are done before deploying, help colleagues with their shift trade needs, be flexible with deployment dates to help your department meet staffing requirements, etc.). Many ex-humanitarians now work in community emergency department leadership—these chairpersons are often very willing to explore innovative staffing models to allow for their staff to serve in humanitarian crises. It is also important to identify a humanitarian organization that will support this model, and engage them in discussions regarding your scheduling constraints early.

### Humanitarian careers in academic EM

International emergency medicine is a recognized subspecialty of academic EM, with formal sections in the Society of Academic Emergency Medicine,^30^ the American College of Emergency Physicians,^31^ and multiple recognized, non-ACGME accredited fellowships.^32^ Academic international EM allows EPs to engage in humanitarian aid and research related to humanitarian aid while maintaining an academic home in the U.S. Academic EPs have been at the forefront of the movement to ensure high-impact, evidence-based humanitarian aid and population-based public health research in humanitarian crises. Several academic institutions have well-established academic EM divisions engaged in multidisciplinary field research at their institutions.

EPs with experience in public health research and health systems development and analysis can often engage in multiple short-term deployments over several years, collaborating with organizations that operate in conflict-affected settings to strengthen their response, measure impact, investigate health and human rights violations, or develop an evidence base for improved health programming. EPs interested in this type of work and a career in academic EM can consider international EM fellowships mentioned above. These fellowships typically involve project fieldwork, clinical practice in an academic center, and the opportunity for a master’s degree in public health. International EM fellowship graduates work in humanitarian and other global health organizations, the Centers for Disease Control, U.N.-based groups such as UNICEF and WHO, as well as in academic EM centers.

### Supporting your colleagues/fundraising

EPs interested in assisting the humanitarian aid effort can lead highly successful fundraising efforts and awareness events within their communities, religious organizations, and hospital systems. Aid can then be donated to organizations with active aid efforts in the affected area. Consider working a shift and donating the proceeds to an agency delivering aid, and asking colleagues to do the same. As mentioned above, a relatively small amount of cash can go a long way. Do keep in mind that *cash* is always the most helpful resource in any setting—as donated medications, supplies and clothing are often expensive to transport and not appropriate for local use on arrival.^32^ Many EPs with significant humanitarian aid experience will need their colleagues’ help to deploy to a crisis; supporting an experienced colleague in their deployment by taking a shift is a tremendous help.

Finally, it is our responsibility to educate our colleagues on the provision of responsible humanitarian aid.

## CONCLUSION

Still haunted by the images of the earthquake, you arrive for your 7am shift. Your post-overnight colleague had previously worked with MSF and just became aware of the earthquake. You speak to him about your desire to help and ask him how you can get involved.

You have a conversation about many of the issues discussed above, and ultimately decide that you will help lead an effort to clear your colleague’s schedule so he may travel, and raise funds to support MSF in Haiti. You also ask him to let you know when the next MSF recruitment meeting is in your city.

EPs have the flexibility, multi-disciplinary clinical skills, and exposure to intensive and chaotic work environments that allow them to provide high-impact, meaningful aid to crisis-affected populations. However, it is crucial that EPs carefully consider the impact and potential harm of any humanitarian deployment and consider alternative mechanisms to providing on-the-ground aid. EPs interested in engaging in meaningful humanitarian response should take coursework to learn about existing coordination systems and health issues they are likely to encounter in crisis settings. Deployments should be prepared for well in advance and undertaken with reputable humanitarian organizations. Medical students and residents should not engage in crisis response, and on occasion, the donation of money that would otherwise be spent on an ill-advised deployment may be more responsibly and effectively given to experienced organizations.

## Figures and Tables

**Table:** Field texts for health issues in crises

Brent A, Davidson R, Seale A. *Oxford Handbook of Tropical Medicine*, Oxford, England, 2014, Oxford University Press.
Beeching NJ, Gill GV: *Lecture Notes on Tropical Medicine*, Oxford, England, 2014, Blackwell Publishing Ltd.
Médecins Sans Frontières Reference Books (various): refbooks.msf.org.
Lampard B, et al. Global Humanitarian Medicine and Disaster Relief. In: *Auerbach’s Wilderness Medicine*.7th ed. Philadelphia, PA. Elsevier; 2012:1893–1928
Perrin P: H.E.L.P. *Public Health Course in the Management of Humanitarian Aid*, Geneva, Switzerland, 2001, International Committee of the Red Cross.
Connolly MA, editor: *Communicable Disease Control in Emergencies: A Field Manual*, Geneva, Switzerland, 2005, World Health Organization: who.int/iris/bitstream/10665/96340/1/92415 46166_eng.pdf.
WHO Technical Report Series 985: *The Selection and Use of Essential Medicines: Report of the WHO Expert Committee*, 2013, Geneva, Switzerland, 2014, World Health Organization: who.int/iris/bitstream/10665/112729/1/WHO_TRS_985_eng.pdfs/.
David Werner, Carol Thurman, and Jane Maxwell: *Where There Is No Doctor: A Village Health Care Handbook*, 2013, Hesperian Foundation: hesperian.org.
Murray Dickson: *Where There Is No Dentist*, 2012, Hesperian Foundation: hesperian.org.
Maurice King: *Primary Surgery, Volumes I, II, III*, Oxford Medical Publication: primary-surgery.org/start.html.
*Surgical Care at the District Hospital: The WHO Manual*: who.int/surgery/publications/imeesc/en/index.html.
Giannou C, Baldan M, War Surgery, May 2010, ICRC: icrc.org/eng/assets/les/other/icrc-002-0973.pdf.
Michael B. Dobson: *Anesthesia at the District Hospital*, World Health Organization: who.int/iris/handle/10665/42193.pdf.

* From Lampard B, et al. Global Humanitarian Medicine and Disaster Relief. In: *Auerbach’s Wilderness Medicine*.7th ed. Philadelphia, PA. Elsevier; 2012:1893–1928.
